# Global computational mutagenesis of domain structures associated with inherited eye disease

**DOI:** 10.1038/s41598-019-39905-9

**Published:** 2019-03-06

**Authors:** Francisca Wood Ortiz, Yuri V. Sergeev

**Affiliations:** Ophthalmic Genetics and Visual Function Branch, National Eye Institute, National Institutes of Health, Bethesda, MD USA

## Abstract

Multidomain proteins account for 70% of the eukaryotic proteome. In genetic disease, multidomain proteins are often affected by numerous mutations, but the effects of these mutations on protein stability and their roles in genetic disease are not well understood. Here, we analyzed protein globular domains to understand how genetic mutations affect the stability of multidomain proteins in inherited disease. In total, 291 domain atomic structures from nine multidomain proteins were modeled by homology, equilibrated using molecular dynamics in water, and subjected to global computational mutagenesis. The domains were separated into 7 groups based on protein fold homology. Mutation propensities within each group of domains were then averaged to select residues critical for domain fold stability. The consensus derived from the sequence alignment shows that the critical residues determined by global mutagenesis are conserved within each group. From this analysis, we concluded that 80% of known disease-related genetic variants are associated with critical residues and are expected to have significant destabilizing effects on domain structure. Our work provides an *in silico* quantification of protein stability and could help to analyze the complex relationship among missense mutations, multidomain protein stability, and disease phenotypes in inherited eye disease.

## Introduction

The amino acid sequence of a protein encodes its three-dimensional structure and its biological function^1^. The polypeptide chain undergoes a stochastic folding process through the many possible conformations accessible until the most thermodynamically stable structure is achieved. The native state of proteins generally corresponds to this structure. When the energy surface or ‘landscape’ has the correct shape, only a small number of the available conformations must be sampled^2^. The energy landscape, encoded by the amino acid sequence, can consequently change in response to changes in the sequence. These disturbances in the energy landscape often result in an unfolded or partially folded protein structure, and it follows that these structural changes translate into functional changes^3^.

Protein domains, described as compact globular units usually containing 50–150 amino acids, are individually folded to maintain proper structure and function^4^. Multiple domains provide structural and functional plasticity in single-domain proteins, and multidomain proteins are thought to have higher stability within the proteome^5^. Genome analyses show that over 70% of eukaryotic proteins consist of multiple domains^6,7^, but large proteins are difficult to study experimentally. Determination of 3D structure for multidomain proteins presents a number of challenges due to their large molecular size^8^, which can span 1000–5000 amino acid residues each^4^.

In human inherited disease, genetic mutations may affect the stability of domain structures. Although the exact role of missense mutations in inherited disease is not well understood, these genetic mutations can disrupt the energy landscape and result in protein misfolding and loss of protein stability^9^. Many inherited diseases are linked to protein misfolding resulting from missense mutations, which are believed to disrupt the physicochemical properties of proteins, such as charge, hydrophobicity, and geometry^10^. Although a precise link between genetic mutations and disease phenotype is difficult to establish, it has been shown computationally that the perturbations caused by genetic mutations modeled at the atomic level of protein structure were associated with patients’ b-wave electroretinogram (ERG) amplitudes, as demonstrated for X-linked retinoschisis^11,12^.

An *in silico* global mutation screen was developed to evaluate the effect of missense mutations on protein stability^13–15^. This approach calculates an unfolding propensity for each missense mutation, which is derived from the changes in Gibbs free energy between the wild-type and mutant protein structures, to describe the effect of the mutation on protein stability. The ‘foldability’ parameter is a new parameter that identifies residues in the protein structure that could play a role in protein destabilization^13^. Residues with high foldabilities lead to protein destabilization, indicating the essential role of each residue in maintaining proper protein folding and stability. In contrast, residues with low foldabilities will typically maintain the proper protein fold despite the presence of a mutation. Residues with the highest foldabilities are referred to as “critical” residues for the critical role they play in proper protein folding^15^. Following the principle that many inherited diseases may be caused by misfolding due to missense mutations, residues described as critical are expected to coincide with residues associated with inherited disease. Hence, the unfolding mutation screen (UMS) serves as a tool to analyze the complex relationship among missense mutations, protein folding, and disease. The results of this study can be found at the Ocular Proteome website (https://neicommons.nei.nih.gov/#/proteome).

Currently, the analysis of genetic mutation effects on multidomain proteins is hampered by the absence of information on the complete 3-dimensional structures of these proteins. However, as protein domains behave as autonomous folding units^7^, the effect of mutations in a particular domain could be considered as a perturbation of protein stability and could be evaluated for each individual domain. Therefore, due to the lack of defined multidomain protein structure, an algorithm was developed to evaluate the stability of each protein domain.

Here, 9 multidomain proteins associated with different inherited eye diseases were studied. In total, 291 protein domains were individually subjected to UMS, and structural alignments of homologous domains were used to average the unfolding data into a single unfolding matrix describing each domain type. The averaged unfolding matrix was used to calculate foldability, and critical residues were identified at high-foldability positions, which occurred across all domains. The analysis showed that 83% of disease-causing mutations (retrieved from the Human Gene Mutation Database, HGMD) that resulted in large magnitudes of destabilization (those with predicted unfolding propensities above 0.9) occurred in critical residues with high foldabilities, suggesting that residues critical for maintaining proper domain folding could also be critical for stability of the multidomain protein. Additionally, the sequence alignment of each domain set studied reveals conservation of residues described as critical for protein stability across the domains of various proteins, highlighting the importance of these residues for protein structure.

## Results

**Figure 1:**
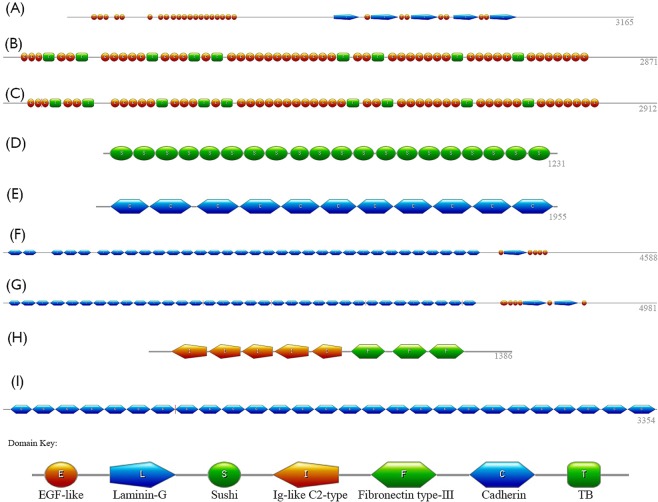
Domain structures of nine proteins in inherited eye disease. Each panel represents the sequence of domain structures, which varies greatly for each protein, and the domain key can be found at the bottom of the figure. EYS in panel A contains 27 EGF-like (orange circles) and 5 laminin-G domains (blue pentagons). FBN1 and FBN2 in panels B and C, respectively, each contain 47 EGF-like and 9 TB domains (green squares). Panel D shows the 20 sushi domains (green circles) of CFH. Panel E shows the 11 cadherin domains (blue hexagons) of PCDH15. FAT1, in panel F, contains 33 cadherin domains, 5 EGF-like domains and 1 laminin-G domain, while panel G contains the 34 cadherin, 6 EGF-like and 2 laminin-G domains of FAT4. Panel H shows the Ig-like C2-type (orange pentagon) and fibronectin type-III (green hexagon) domains of ROBO3. Panel I show the 27 cadherin domains of CDH23.

Protein domain stability was analyzed for nine proteins, eyes shut homolog (EYS), Fibrillin-1 (FBN1), Fibrillin-2 (FBN2), complement factor H (CFH), protocadherin-15 (PCDH15), protocadherin fat 1 (FAT1), protocadherin fat 4 (FAT4), roundabout homolog 3 (ROBO3), and cadherin-23 (CDH23) (Table 1). Genetic mutations in these proteins cause genetic eye diseases such as retinitis pigmentosa, Marfan syndrome, and age-related macular degeneration. The structural organization of these multidomain proteins is shown schematically in Fig. 1.

**Table 1:** Protein domain information for proteins in inherited eye disease.

Protein	Diseases	UniProt ID	Protein residue	Structural domains	HGMD mutation
Eyes Shut Homolog, EYS	RP, LCA	Q5T1H1	3165	EGF-like (27) Laminin (5)	155
Fibrillin-1, FBN1	Marfan Syndrome	P35555	2871	EGF-like (47) TB (9)	1580
Fibrillin-2, FBN2	Contractural arachnodactyly	P35556	2912	EGF-like (47) TB (9)	64
Complement Factor H, CFH	Age-related macular degeneration	P08603	1231	Sushi (20)	219
Protocadherin-15, PCDH15	Usher syndrome	Q96QU1	1955	Cadherin (11)	60
Protocadherin, FAT1	Head and neck squamous cell carcinoma	Q14517	4588	Cadherin (33) EGF-like (5) Laminin (1)	20
Protocadherin, FAT4	Hennekam syndrome	Q6V0I7	4981	Cadherin (34) EGF-like (6) Laminin (2)	23
Roundabout homolog 3, ROBO3	Gaze palsy	Q96MS0	1386	IG-like C2-type (5) Fibronectin (3)	21
Cadherin-23, CDH23	Usher syndrome	Q9H251	3354	Cadherin (27)	234

The nine proteins described are identified by protein name, UniProt accession number, polypeptide length, number and types of structural domains, and number of missense mutations retrieved from the Human Gene Mutation Database, HGMD.

The proteins were split into individual domains and then divided into 7 groups by homology. These domains were epidermal growth factor-like (EGF-like), laminin globular (laminin-G), sushi, immunoglobulin-like C2-type (Ig-like C2-type), fibronectin type-III, cadherin, and transforming growth factor-beta (TB). In total, the 291 protein domain structures were each individually homology-modeled (Supplemental Table S1), equilibrated using 2 ns molecular dynamics in water to achieve better domain stereochemistry, and subjected to global computational mutagenesis using the UMS^13^ to evaluate the effect of mutations on their protein stability. Panels A-I images were prepared  using the SMART (http://smart.embl-heidelberg.de/).

The disease-mutation data for each protein were then quantified using unfolding propensities, which were retrieved for each mutation from its appropriate unfolding matrix. These data revealed that most inherited-disease-causing mutations are associated with large destabilizing effects (unfolding above 0.9), consistent with the literature^16^. To quantify the overall pattern of mutation changes between similar domains, the unfolding propensities of homologous domains within each protein were averaged to filter out noise related to structural domain variations. This procedure isolates residues that have a higher propensity for protein structure destabilization and are therefore critical for protein stability. Mapping of disease-causing mutations and identification of critical residues shows correspondence between residues identified as critical *in silico* and residues associated with disease-causing mutations. Mutations in critical residues are associated with a wide range of inherited diseases, revealing the critical roles of these residues in protein structure and stability. Furthermore, the sequence alignment of each domain group reveals conservation of critical residues, with 50% of all conserved residues predicted to be critical residues. These results support a critical stability framework of residues that is conserved across domains to provide essential components of protein structure and stability.

### Quality of domain structures

Domain structures were modeled as described in the Methods section to ensure viable, stable structures with proper stereochemistry. To assess the quality of the improved models, they were subjected to an internal control designed to verify the quality of the side-chain rotamers of the model^13^, and Ramachandran plots were produced to verify plausible dihedral angles^17^.

An internal control was used to create self-mutated structures for each residue in the protein sequence. Quality models subjected to the internal control were expected to produce confidence intervals centered on 0.5 with small p-values (~10^−2^). Supplemental Table S2 contains the internal control values for the domains modeled, and Supplemental Fig. S1 shows the distribution of p-values produced by the internal control. More than 75% of the domain models produced p-values of 0.05 or smaller. Potentially, the quality of these domains individually could be improved using longer periods of molecular dynamics. However, this work would require significant additional computational time to perform this kind of simulation for all domains. Averaging the domain propensities offsets this shortcoming and serves to improve the overall descriptors of domain structure stability.

**Figure 2:**
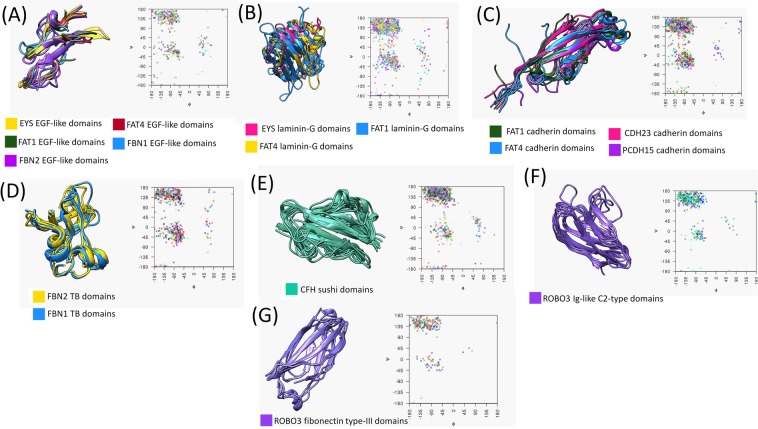
Quality of domain structures. Panels A through F correspond to the EGF-like, laminin-G, cadherin, TB, sushi, Ig-like C2-type, and fibronectin type-III domains, respectively. The column on the left contains structurally superimposed domains shown by ribbon structures. Similar structures within each domain type are distinguished by protein color. The axes on the right of each panel contain the Ramachandran plots for each domain set, with arbitrary colors representing different domain models. For the EGF-like (**A**) and cadherin (**C**) domains, only 15 structures were selected to show in the Ramachandran plot. All domain structures of laminin-G (**B**), TB (**D**), sushi (**E**), Ig-like C2-type (**F**) and fibronectin type-III (**G**) were superimposed.

The structures were further validated using Ramachandran plots to illustrate the distribution of backbone dihedral angle data, and the plots are included in Fig. 2 for the 7 domain groups studied. The Ramachandran plots shown have concentrated backbone dihedral angles in the energetically allowed region. Additionally, the Ramachandran plots in Fig. 2 are accompanied by structurally aligned domain structures to show the consistency and reliability of the structures built using homology modeling.

Due to computing limitations brought about by Chimera, only a subset of 15 domains from the cadherin and EGF-like domains could be studied using structural alignment. Therefore, only 15 domains were shown in Fig. 2 for each group, to allow better visualization of domain superposition. These 15 domains were selected at random to reduce bias. To ensure the 15-domain representative sample was a valid representation of the whole set, 5 different 15-domain subsets of EGF-like domains were structurally aligned. The alignments produced by these subsets were compared upon their primary structure alignment and secondary structure alignment. The alignment of these was determined to be similar enough across subsets to validate the methodology proposed in the manuscript. Once this approach was validated, 15-domain subsets of the EGF-like and cadherin domains were selected for this study. Thus, the Ramachandran plots, internal control, and structural alignment of each domain set validated our modeled structures.

### Disease-related mutant variants

Considering that many inherited human disorders are believed to arise from protein destabilization^16^, the effect of disease-causing mutations on domain stability was quantified. A number of disease-causing missense mutations were retrieved from the HGMD (http://www.hgmd.cf.ac.uk/) for each of the nine multidomain proteins (Table 2). Mutations that did not map to the individual protein domains were excluded from our analysis. The number of retrieved missense mutations is shown in Table 1. For each mutation, the propensity of domain destabilization was obtained from the unfolding propensity data matrix produced by the UMS. The average unfolding fraction of all missense mutations ranged between 0.59 ± 0.26 and 0.82 ± 0.22. Mutations with predicted unfolding fractions above 0.9 were categorized as severe-destabilization variants. Medium- and low-destabilization mutations resulted in propensities below 0.9.

**Table 2:** Disease-causing missense mutations with critical roles in protein stability.

Protein	Retrieved mutations	Missense mutations mapped onto structural domains	Unfolding fraction per domain	Number of mutations with critical roles predicted *in silico*
EYS	155	101	0.78 ± 0.26	42 (42%)
FBN1	1580	1451	0.75 ± 0.22	834 (57%)
FBN2	64	60	0.70 ± 0.27	27 (45%)
CFH	219	214	0.71 ± 0.24	70 (33%)
PCDH15	60	46	0.70 ± 0.30	11 (24%)
FAT1	20	15	0.62 ± 0.24	3 (20%)
FAT4	23	23	0.71 ± 0.28	3 (13%)
ROBO3	21	19	0.82 ± 0.22	9 (47%)
CDH23	234	218	0.59 ± 0.26	29 (13%)

From left to right: protein name; number of mutations retrieved from HGMD; number and average unfolding fraction of disease-causing mutations associated with structural domains, and number and percentage of HGMD mutations with critical roles (unfolding fraction > 0.9).

We found that the average unfolding propensity due to disease-causing missense mutations for all proteins was 0.71 ± 0.25, a value that corresponds to a medium-level destabilizing effect. The list of disease-causing mutations was further filtered by selection of mutations with high degrees of unfolding (>0.9). The total number of disease-related mutations with predicted severe destabilization effects on domain structure ranged from 13% (FAT4) to 57% (FBN1) and was on average 33%.

The data show that 82% of all severe-destabilization mutations occur in residues considered critical for protein structure. The percent of severe mutations mapping to critical residues ranged from 64% to 100%, revealing a correlation between the residues implicated in disease and those believed to be critical for the structure of individual domains and the whole protein. Moreover, on average, critical residues associated with disease-causing mutations had foldability scores of approximately 14.

**Figure 3:**
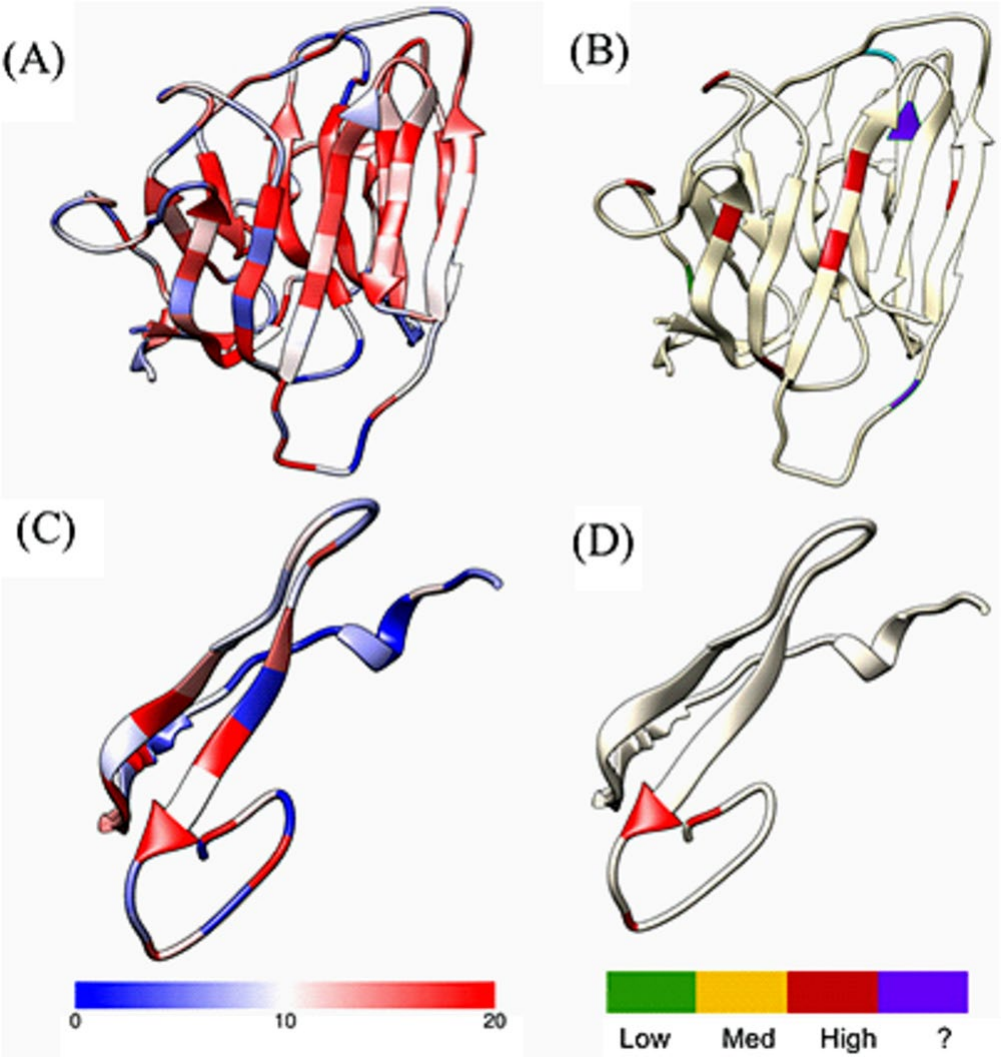
Laminin-G and EGF-like domains of EYS showing similar locations of residues labeled by foldability and disease-related mutations. The laminin-G domain (**A**,**B**) and EGF-like domain (**C**,**D**) of the EYS protein are colored by foldability (left) and the unfolding parameters of disease-causing mutations (right). The foldability scale ranges from 0 to 20, with low-foldability residues shown in blue and high-foldability residues shown in red. The tan residues on the right structure are not associated with any disease-causing mutations, while green, yellow, and red residues correspond to low-, medium-, and high-destabilization mutations. The unfolding parameter ranges from 0 to 1. Mutations colored in purple do not have an associated unfolding propensity.

In Fig. 3, a laminin-G domain (top: A and B) and an EGF-like domain (bottom: C and D) of EYS are shown. The structures are colored by foldability (left) and by severity of disease-causing mutations (right). The mutations identified on the right are colored by the corresponding unfolding propensity. On the left, high-foldability residues are red. Overall, 95% of severe mutations occurred in high-foldability residues (Table 2). Of the 155 known disease-causing mutations, 34% occur in EGF-like domains, and 32% occur in laminin-G domains. Additionally, Supplemental Figs S2 through S6 contain instances of each domain type for ROBO3 (S2), CFH (S3), FBN1 and FBN2 (S4), FAT1 and FAT4 (S5), and CDH23 and PCDH15 (S6). The structures are colored by foldability and disease-causing mutations, and these results support our findings that severe mutations are associated with high-foldability residues in many cases.

The identification of protein missense mutations associated with disease phenotypes showcases the relationship between protein residues associated with disease and individual domain residues that are believed to be critical for domain stability. The results above suggest that stabilizing protein structure residues are critical for maintaining proper folding in individual protein domains.

### Critical residues are conserved across homologous domains

The foldability of identically conserved and similarly conserved residues was examined to assess the relationship between residue conservation and foldability. Supplemental Table S3 and Figure S7 show that 52% of all conserved residues in the seven domain groups were considered critical. By contrast, only 34% of all nonconserved residues in the domain sets were described as critical.

**Figure 4:**
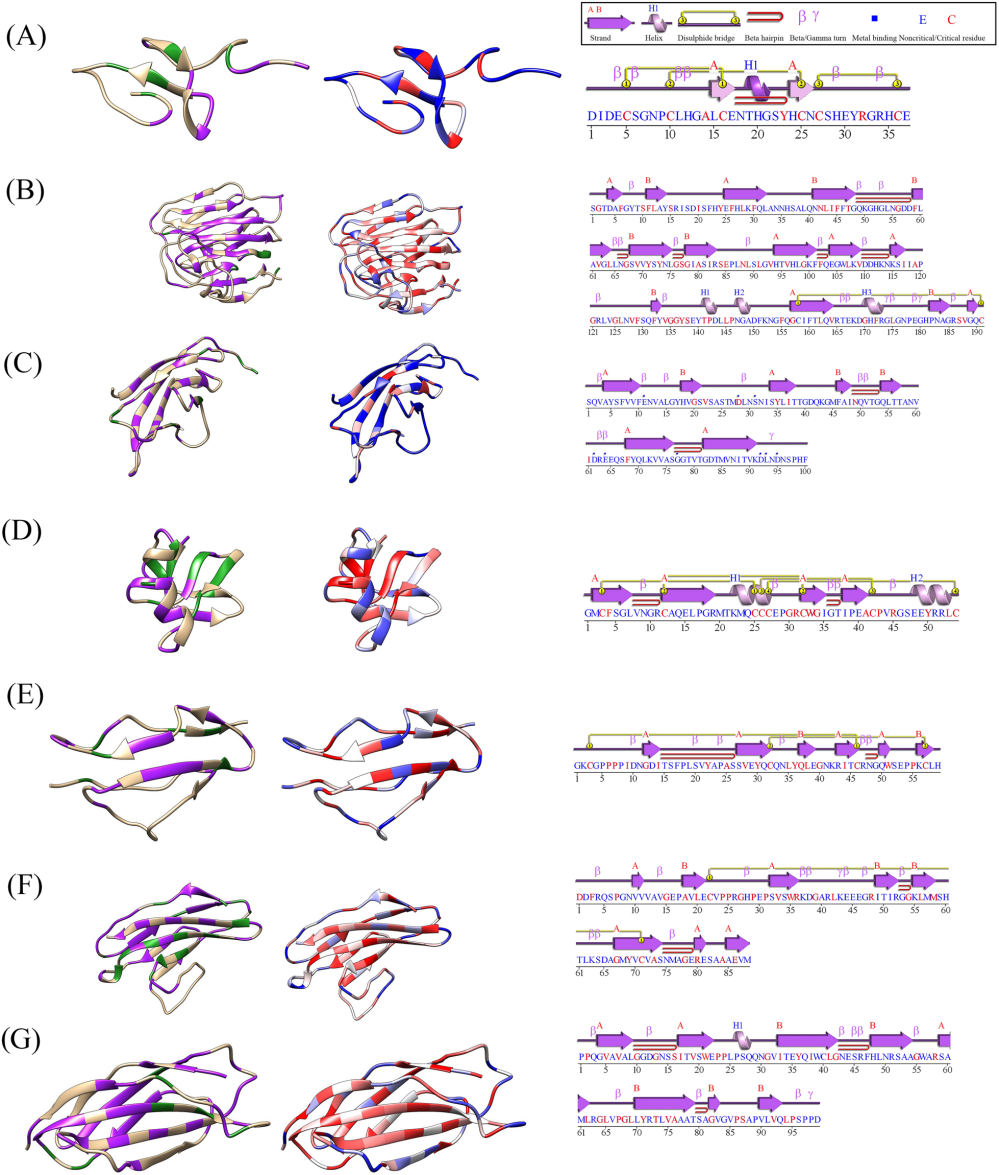
Domains represented as ribbon structures showing conserved residues (left) foldability of residues (middle), and domain secondary structure components (right). The ribbon structures in the first column highlight nonconserved residues (tan), identically conserved residues (green), and similarly conserved residues (purple). The ribbon structures in the center column are colored by a foldability gradient ranging from blue to white to red; high-foldability residues are in red, and low-foldability residues are in blue. The last column contains secondary structure component plots with motifs and disulfide bonds, as well as the domain sequence with critical residues highlighted in red. The panels show an EGF-like domain of FBN1 (**A**), a laminin-G domain of EYS (**B**), a cadherin domain of CDH23 (**C**), a TB domain of FBN2 (**D**), a sushi domain of CFH (**E**), and an Ig-like C2-type (**F**) and a fibronectin type-III domain of ROBO3 (**G**).

In Fig. 4, an example from each group of domains is shown colored by residue conservation (left) and foldability (middle). Each domain sequence is also represented as a secondary structure plot (right), highlighting critical residues in the domain sequence that correspond to secondary structure components. Conserved residues in EGF-like domains (Fig. 4A) were found to have high foldability in 41% of cases. The secondary structure components show a critical role for six cysteine residues, which form disulfide bridges. The conserved residues in laminin-G domains were high-foldability residues in 56% of cases (Fig. 4B). A single disulfide bridge is present in the domain structure of laminin-G, and the cysteine residues that form this bridge are also critical. The alignment of 105 cadherin domains revealed that 49% of conserved residues were also high-foldability residues (Fig. 4C). The TB domain alignment showed that 45% of conserved residues were high-foldability residues (Fig. 4D); the secondary structure components show four disulfide bridges, and all the cysteine residues involved are considered critical. The conserved residues of the sushi domains of CFH had 57% correspondence with high-foldability residues (Fig. 4E), and the cysteine residues involved in the two disulfide bridges of the domain structure were all considered critical. The conserved residues of the Ig-like C2-type (Fig. 4F) domains of ROBO3 were associated with high foldability in 56% of cases, while the conserved residues of the fibronectin type-III domains (Fig. 4G) of ROBO3 were associated with high foldability in 62% of cases. The disulfide bridge present in the Ig-like C2-type domain structure corresponded to critical cysteine residues. Table 3 shows the differences in average foldabilities of conserved and nonconserved residues for each domain type.

**Table 3:** Average foldability of conserved and nonconserved residues of each domain type.

Domain	Conserved residues	Nonconserved residues
EGF-like	8.77	8.72
Laminin-G	10.80	9.55
Cadherin	9.43	8.39
TB	9.60	7.58
Sushi	11.43	9.31
Ig-like C2-type	10.16	10.37
Fibronectin type-III	11.21	9.77

**Figure 5:**
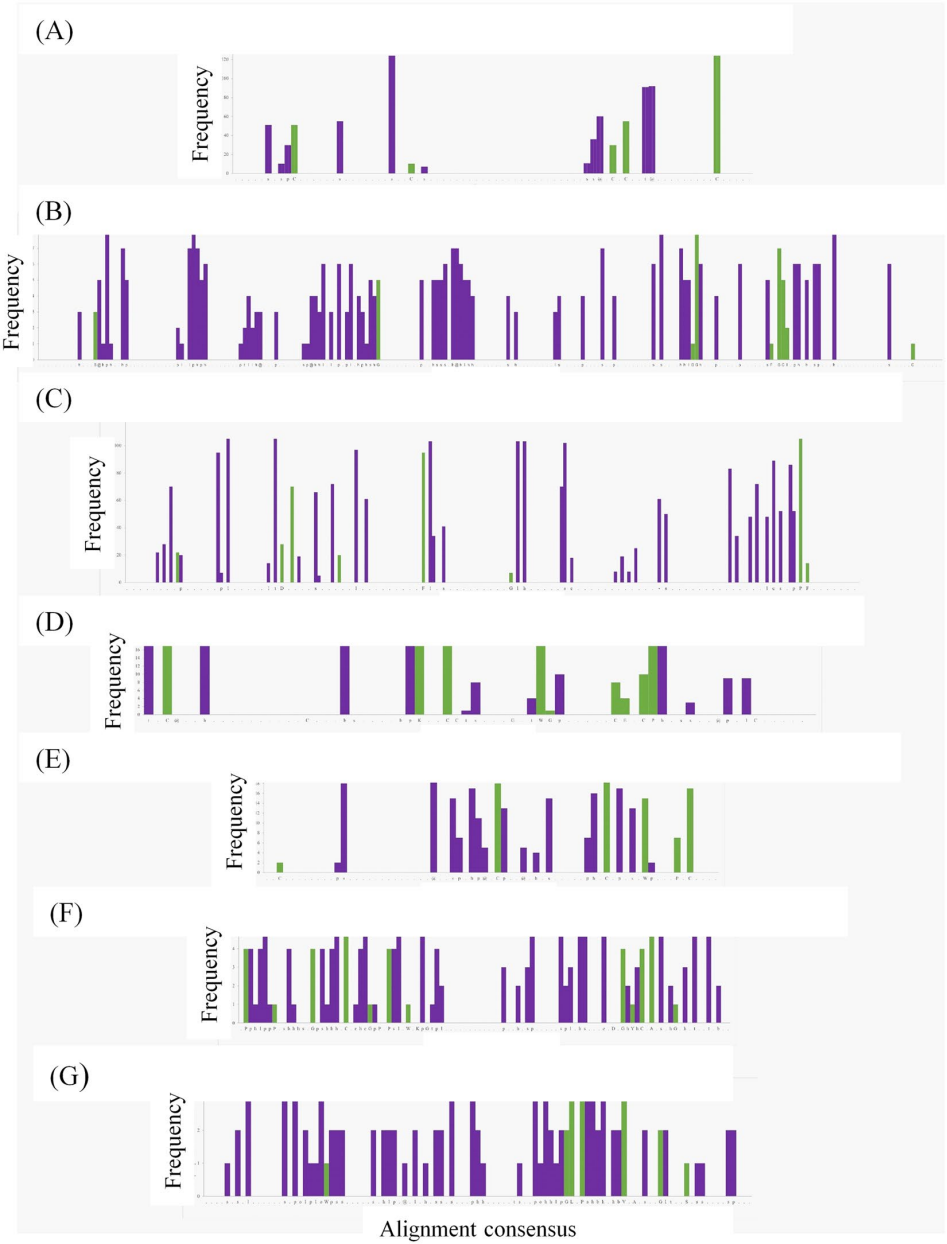
Frequency of critical residues at sequence alignment positions in groups of homologous domains. Panels show the count of critical residues which occur at all sequence alignment points of EGF-like (Panel A), laminin-G (Panel B), cadherin (Panel C), TB (Panel D), Sushi (Panel E), Ig-like C2-type (Panel F), and fibronectin type-III (Panel G) domains, respectively. The alignment consensus can be found along the x-axis, for which a corresponding key can be found in the Supplemental Fig. S7. Frequency counts of critical residues are shown in green for identically-conserved residue alignment points, and in purple for similarly-conserved residue alignment points.

Bar graphs highlighting identically conserved residues and similarly conserved residues over the length of the domain sequence are shown for each domain in Fig. 5. Figure 5A shows the alignment consensus of the 132 EGF-like domains. Overall, 40% of conserved residues were considered critical, while only 25% of nonconserved residues were critical. Figure 5B contains the consensus of the 8 aligned laminin-G domains; 57% of conserved residues were described as critical, and only 32% of nonconserved residues were categorized as critical residues. Figure 5C shows the consensus of the alignment of 110 cadherin domains present in the proteins PCDH15, FAT1, FAT4, CDH23, and CDH3. The consensus showed higher conservation of nonpolar residues towards the middle of the sequence. Aligned residues were considered critical in 45% of cases, and only 39% of nonconserved residues were considered critical. The 18 TB domains of FBN1 and FBN2 were aligned, and 45% of aligned residues of TB domains were also described as critical domains; only 23% of nonconserved residues were described as critical. The alignment consensus of CFH can be found in Fig. 5E. Of all aligned residues, 60% were considered critical. Lastly, the alignment consensus of Ig-like C2-type domains and fibronectin type-III domains from ROBO3 can be found in Fig. 5F,G, respectively. For Ig-like C2-type domains, conserved residues were critical in 52% of cases, and nonconserved residues were critical in 47% of cases. Conserved residues of fibronectin type-III domains had 62% correspondence with critical residues, while nonconserved residues were critical in only 30% of cases. Overall, although the percentage of conserved critical residues for each domain set varied greatly, critical residues were more often associated with conserved residues.

## Discussion

In this work, we sought to find determinants of multidomain protein stability. Multidomain protein structure estimation has remained elusive due to the shortage of suitable experimental and computational methods designed to handle such large proteins. The lack of defined structures for multidomain proteins hinders our ability to understand the relationship between protein stability and disease. Using *in silico* methods, we developed an add-on to the previously published UMS^14^. Nearly 300 domains from 9 multidomain proteins associated with inherited eye disease were homology-modeled and subjected to global computational mutagenesis. This mutagenesis produced unfolding propensity data for all domains studied and a measure of protein stability derived from domain stability for proteins that have otherwise been largely unstudied.

The multidomain UMS identifies the effects of all possible missense mutations on globular domain structures and defining a foldability parameter allows us to identify residues that provide critical stability for proper domain folding. The method developed here uses free energy measures to quantify domain stability (as an unfolding propensity) in response to genetic perturbations, and these descriptors of domain stability are integrated to describe multidomain protein stability. Each domain was subjected to the UMS, which produces unfolding propensity data and stores it in a 2D matrix. Unfolding data matrices for sets of homologous domains of each protein were averaged to obtain consensus patterns of domain stability and filter noise associated with structural dissimilarities and modeling errors. A foldability parameter was derived from the unfolding propensities as a measure of the sensitivity of a residue to missense mutations; a high foldability parameter would correspond to a residue that, when mutated, causes significant destabilizing effects on the domain structure. Residues that are consistently critical for domain structure across all homologous domains were identified. Amino acid residues with high foldabilities across most of each homologous protein domain were determined to be residues critical for protein stability.

The determination of protein stability allowed us to investigate the relationship between protein stability and disease-causing mutations. Disease-related mutations were described by the associated unfolding values to gain an understanding of the effect of disease-related missense mutations on the stability of the domain and subsequently, the entire protein. The proteins described were found to have missense mutations, which resulted, on average, in a destabilizing effect measured as 0.7 unfolding. The distribution of unfolding propensities shows that only ~30% of disease-causing mutations have ‘severe’ destabilizing effects on domain structure, while the remaining 70% of mutations had primarily destabilizing effects.

The sequence alignment produced for each set of domains reveals that a high percentage of conserved residues are considered critical. The data show that nearly 50% of conserved residues are critical for each domain, while nonconserved residues are described as critical in only 30% of cases. These results suggest an apparent relationship between conserved residues and those acting as determinants of protein stability. In part, this observation agrees with our previous results on strong correlation between the protein sequence conservation index and foldability determined for 9 eye disease-related proteins^15,18^. However, a comparison of average foldabilities of conserved and nonconserved residues of each domain type as shown in a Table 3 do not support this result. Although the average foldability of nonconserved residues was found to be lower than the average foldability of conserved residues for 5 domains, the differences do not show the same trend for EGF-like domain and Ig-like domain.

Protein polypeptides fold to produce a native protein structure within approximately 1 to 30 ms^19^, but simulations of protein folding for such lengths are computationally expensive. At 2 ns, only very early events of protein destabilization can be modeled. Although these very short simulations may not represent a model for the folding-unfolding process, we assessed the proteins using an internal control designed as a measure of protein model quality. The distribution of p-values from each domain internal control, shown in Fig. S1, includes the percentage of domains with p-values less than 10^−2^. Although a well-refined model should have a p-value with a magnitude of 10^−5^ or better, domains with p-values smaller than 0.05 were still considered attainable structures in our application. In addition, Ramachandran plots of homologous domains showed dihedral angles primarily in energetically allowed regions, which allowed us to appraise these structures as representative models of domain structures.

Protein domain structures and functions are, to some extent, conserved between homologous domains in different proteins. The seven homologous domains from the 9 selected proteins were studied to inspect which residues are mostly conserved across proteins, as well as the relationship between conserved residues and critical residues. For each domain, conserved residues were also classified as critical residues. Over 600 disease-causing mutations were associated with severe destabilization effects on domain structures, and approximately 30% of the disease-causing mutations occurred in conserved residues that were considered critical across all proteins studied. These results highlight the importance of residues described as critical for proper protein folding across many different protein families and establishes their importance for healthy phenotypes.

An evident restriction of our current approach is the lack of domain-domain interactions that may favor stability. It is known that in multidomain proteins, domain-domain interactions aid in stabilizing domains^5^. However, since it is also known that only a fraction of all domain residues are involved in interactions with other domains^7^, our approach remains valid. In the future, we hope to develop a multidomain UMS application that incorporates stability derived from domain assembly, since correctly arranged domain structures are often crucial for a full understanding of the functions of multidomain proteins^20^.

Within eukaryotes family, about 67% are multidomain proteins^21^. Multi-domain proteins vary significantly in molecular weight. For example, proteins analyzed in this work (Fig. 1) are changing their molecular weight from 139 kDa (CFH) to 507 kDa (FAT1). The largest multi-domain protein, titin from human muscle has 34,000 residues in a protein sequence, the molecular weight of 3816 kDa, and includes about 132 fibronectin type III and 152 Ig-type domains (Uniprot, # Q8WZ42). Many multi-domain proteins are difficult for the structural analysis and their full-length protein atomic structures currently not available.

Protein databases contains structures of many small domains, which are building blocks of multi-domain proteins. This information could be used for the computational analysis of the domain protein stability. Here we assume that multi-domain protein is a combination of domains, each of these is an independent folding unit. The assumption about the domain independence could be in question if there is a wide-range interaction between neighbor domains within a chain. Indeed, recently it was shown that domain folding for some proteins might depend on inter-domain interface or linker length and flexibility^7^. This consideration is limiting the analysis of single domains and requires the application of global mutagenesis for the analysis of whole multi-domain protein (not for independent folding units). The availability of multi-domain protein atomic model is the only limitation for the global mutagenesis analysis. The analysis is standard for our method and numerous protein structures were already analyzed using this technique (https://neicommons.nei.nih.gov/#/proteomeData).

Two large proteins, FBN1 and FBN2, form fiber-like multi-domain structures^22^, each is composed of 47 EGF-like domains interrupted by 9 TB domains (Fig. 1). From crystal data (PDB files: 2BO2, 2BOU, 2BOX) we might expect small interfaces and very short linkers between domains suggesting independent folding of EGF-like domains that agrees with the analysis the disease-causing mutations^23,24^. TB domains are included in a sequence of protein domains and separated by linkers of 6 and 18 residues at the N- and C-termini from other domains, respectively. This also indicates that folding of TB domains could be independent. Loosely packed small interfaces also are expected for other proteins such as Laminin-G domain (PDB file: 1H30), Cadherin domains (PDB files: 1L3W,2NCJ, 2MVS), and Sushi domain (PDB file: 2QFG). Two domains, Ig-like type and fibronectin type III domains, are independent folding units^7^.

Individual domains of the multi-domain protein are synthesized consecutively *in vivo* by ribosome. Protein biosysthesis starts from protein N-terminus by a mechanism known as co-translational folding^25^. During protein synthesis, the nascent peptide travels through the polypeptide exit tunnel of the ribosome, which has a length of about 100 Å and covers about 30–40 amino acids of the nascent peptide in a fully-extended conformation^25^. Smaller structures such as secondary structure elements can form within the tunnel. However, larger structural domains can be formed when the protein domain appears from the peptide exit tunnel of the ribosome^26^. Synthesis of whole multi-domain protein might require a significantly longer time (4–16 minutes for the proteins from Table 1) if assume that protein synthesis performed with a rate of 50–300 residues/ min for cell-free systems and somewhat faster *in vivo*^27^. The secondary structure formation and protein domain compaction might require less than 1 s^28^.

The pathogenic effect of a genetic mutation on will destabilize protein domain folding at very first stages of protein synthesis when isolated protein fragment containing the mutation is localized to either a ribosomal tunnel or a tunnel exit (for larger domains). The domain will be relatively ‘isolated’ from other parts of the multi-domain protein and the perturbation caused by mutation could be analyzed computationally.

The automation of the multidomain UMS reduces the need for human labor and has the potential for cloud-based applications. If the domains and unfolding mutation data are prepared in advance, stability information about a protein can be retrieved virtually instantaneously. The data produced in this work are freely available at https://neicommons.nei.nih.gov/#/proteome.

In conclusion, the multidomain UMS pipeline developed in this work allows us to predict residues and regions in multidomain proteins that are critical for protein structure and function. In the future, a knowledge of critical residues will allow us to examine the relationship among mutations in multidomain proteins, effects on protein stability, and disease phenotypes.

## Methods

### Molecular modeling

Protein domain, sequence, and mutation information for 9 multidomain proteins are shown in Table 1. Protein amino acid sequences and domain ranges were automatically retrieved from the UniProt database (http://www.uniprot.org/) using the accession ID numbers and a python script. The domain sequence for each protein can be found in Fig. 1, which shows the variability in domain number and structure of the nine proteins.

Each protein domain was generated by homology using the molecular graphics, modeling and simulation program YASARA^29^. A homology modeling experiment in YASARA uses the target protein sequence to identify possible structural templates by running 3 PSI-BLAST iterations to extract a position specific scoring matrix (PSSM) from UniRef90. The Protein Data Bank (PDB) is then searched for a match, and YASARA builds models for each matched template. For each template, if the alignment is unambiguous, a single model is built. If the alignment is ambiguous, several alternative models may be built. A ‘quality Z-score’ is calculated from the molecular dynamics force field energies, capturing the correctness of the backbone- and side-chain dihedrals as well as packing interactions. The models are ranked by their overall quality Z-scores, and the best parts of each model are combined to obtain a hybrid model, in hopes of synergistically increasing the accuracy.

The obtained 3D atomic structure for each domain was equilibrated using molecular dynamics in water using the standard macro ‘md_runfast’ in YASARA. The macro is optimized with the LINCS algorithm to run accurate molecular dynamics with a ‘fast’ speed setting at a 2 × 2.5 fs time step. Structures are placed in a cubic simulation cell extending 20 Å around the domain structure. The AMBER14 forcefield is used with a periodic cell boundary, and long-range electrostatics used a particle mesh Ewald algorithm with an 8.0 Å distance cutoff. The macro is preset to achieve pH 7.4 and 0.9% NaCl (153 mM) concentration at 25 °C. Sodium and chlorine ions are placed at the locations of the lowest and highest electrostatic potentials until the cell is neutralized. The location of the counter ions does not matter in practice, as they will randomly diffuse away through the simulation^15^. The simulation aims to reach a water density of 0.997 g/l and will adjust the pressure accordingly to obtain the previously stated parameters. The simulation frames are saved every 250 ps. The domain structure is subjected to 2 ns of fast molecular dynamics (‘md_fast.mcr’) until the structure is determined to be feasible. A total of 291 domains were modeled. Supplementary Table S1 shows the templates used to build the structures.

Disease-related missense mutations were retrieved from the HGMD (http://www.hgmd.cf.ac.uk/).

### Global mutagenesis

The domains were then subjected to the UMS to generate unfolding propensities for all possible missense mutations, which were organized into a 2-dimensional matrix^12^. The identity mutation assessment^13^ was applied to these models. In this method, a residue is mutated to itself. Since no significant changes in structure should occur under a self-mutation, the stability and free energy should remain the same. The mean, standard deviation, p-value, and 95% confidence interval of each identity mutation in the protein sequence were calculated to provide a measure of the quality of each domain. The protein domain structures, disease information, results of global computational mutagenesis, and disease-related mutations are available at the Ocular Proteome website (https://neicommons.nei.nih.gov/#/proteome).

### Foldability

Foldability is a parameter first described by McCafferty and Sergeev^12^; this parameter is used to describe the sensitivity of each location to missense mutations. Foldability is calculated for each alignment position to evaluate the frequency of severe mutations for each location through summation of all unfolding propensities greater than 0.9. The foldability scale ranges from 0 to 19, where a foldability of 0 represents an alignment position at which residues can be mutated without significant effects on structure, and 19 represents a residue that results in severe destabilization when mutated.

### Critical residues

Critical residues for each protein were first described by McCafferty and Sergeev^13,14^ as the residues with the highest foldabilities. In our work, foldability is calculated from an aligned ensemble of residues. At each alignment position in the aligned FASTA file (AFASTA), a p-value is calculated using analysis of variance (ANOVA). This p-value quantifies the variability in the unfolding propensities of all residues averaged. Here, critical positions are described as the AFASTA alignment positions with the highest foldability values (>10) and p-values below 0.05. High foldability values identify positions that result in severe protein destabilization, while the accompanying p-value describes the variability at that position. AFASTA alignment positions that invariably result in high destabilization (high foldability, small p-value) are determined to be critical across all domains, creating a critical-residue framework that provides stability across all domains.

### Multidomain UMS algorithm

**Figure 6:**
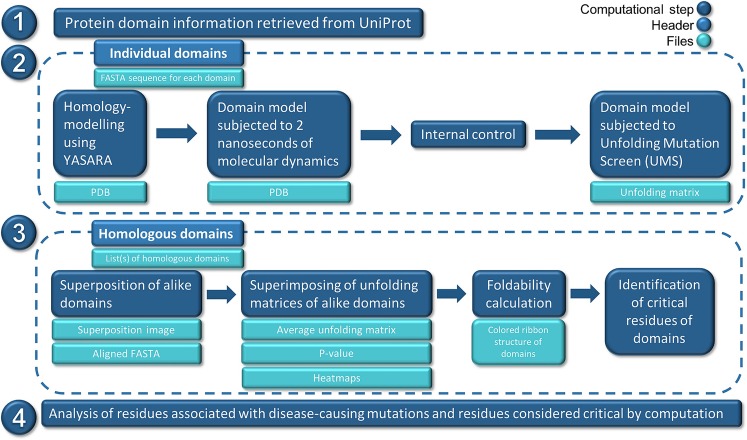
Multidomain UMS pipeline depicting automated processing. (1) Protein domain information is retrieved from UniProt, and (2) domains are homology-modeled, equilibrated in 2 ns of molecular dynamics, compared with an internal control, and subjected to UMS. (3) Sets of homologous domains from each protein are superimposed, and their UMS data are averaged. Foldability and p-values from the averaged UMS data are used to identify critical residues for each domain, and (4) analysis is performed.

A python/bash script was developed to automate the processing of protein domain data. The code is available on request, and the method design is shown in Fig. 6. Given a UniProt accession ID, the multidomain UMS algorithm can retrieve protein domain information, model the domains, and subject them to the UMS (1 and 2 in Fig. 2). Homologous domains are then structurally aligned, and using the structural alignment produced by UCSF Chimera, the unfolding matrices produced by the UMS are averaged to generate a unique unfolding matrix for each domain type. P-values of the averaged unfolding propensities at each alignment position were calculated using ANOVA. The averaged unfolding matrix of each domain type is used to calculate the foldability of the residues comprising the domains. The foldability is written into an attribute file for each domain, which is used to color all domain structures using UCSF Chimera. Critical residues are identified using the criteria described in the Methods.

### Domain superposition

The structures of the homologous domains of each protein were superimposed using UCSF Chimera^30^, and the sequence alignment was output as an AFASTA file based on the structural superposition. To increase the accuracy of critical-residue identification, the unfolding propensities of the aligned residues of each domain were averaged for each domain set. At each AFASTA alignment position, a p-value was calculated from the distinct unfolding propensities of each residue averaged using ANOVA.

Homologous protein domains was aligned using PROMALS3D^31^, a server that constructs alignments for multiple protein sequences. The alignment of each domain set provides an alignment consensus that identifies the aligned residues identical within each domain (identically conserved residues), as well as positions in the domain sequence where similar residues are conserved (similarly conserved residues). The averaging of domain structures removes the noise introduced by each structure and illuminates residues with consistent unfolding effects across domain structures.

## Supplementary information

Supplemental Material

## Data Availability

Ocular proteome protein domain structures and UMS data from this work available at the NEI Commons web-site (https://neicommons.nei.nih.gov/#/proteome).
