# Supplementary material for: Global computational mutagenesis of domain structures associated with inherited eye disease
Source: Sci Rep. 2019 Mar 6;9:3676. doi: 10.1038/s41598-019-39905-9 (PMC6403380; doi:10.1038/s41598-019-39905-9)
Supplement: Supplementary file 1 — Supplemental Material [file 41598_2019_39905_MOESM1_ESM.pdf]

Supplemental Material.

Global computational mutagenesis of domain structures associated with inherited eye disease. Francisca Wood Ortiz, Yuri V. Sergeev

Supplemental Table S1. Templates used to model protein domains. Each protein (column 1) is associated with up to three domain types (column2), for which the templates used to model each domain can be found in column 3. The templates shown can be accessed through RCSB.

Protein	Structural domains	Templates used
EYS	EGF-like	2E26, 5VC1, 5KHX, 1EDM, 5VC1, 1PFX, 5KYO, 5LOV, 5KY3, 1EDM, 3R05, 3QCW, 4D0E, 4YLQ, 4RRX, 30JY, 3SH4, 3NT1
	Laminin-G like	3PVE, 5IK4, 5IK8, 1PZ7, 3SH4, 5MC9, 2WJS
FBN1	EGF-like	2M74, 2WG3, 1UZK, 2W86, 4D03, 2JKH, 5L0V, 2BOU, 1IJQ, 4ZYU, IGL4, 5MWB, 3SOV, 4BDX, 4LOR,
	TB	1UZK, 1APJ, 1UZP, 2W86,
FBN2	EGF-like	2M74, 2YGO, 4BDX, 1EDM, 1UZK, 2W86, 5MS9, 2JKH, 2BOU, 1GL4, 3SOV, 1DX5, 1UZP, 1IJQ, 5L0V, 4D0E, 4XL1,
	TB	1UZK, 2W86, 5MS9, 1APJ, 1UZP,
CFH	Sushi	2WII, 4K12, 4AYI 2V8E, 1OK3, 2XRB, 3J3R, 3R62, 3ZD1, 3R62, 3KZJ, 1OJV, 3KXV, 3ZD1
PCDH15	Cadherin	4APX, 5CYZ, 5T4N, 1ZXK, 3MVS, 5TPK, 4XHZ, 5KJ4, 5DZY, 4ZPM
FAT1	Cadherin	5ERD, 5T4M, 4ZI9, 5TPK, 1ZXK, 4AQA, 2WCP, 4ZPM, 5SZQ, 3MVS, 4OY9, 5SZR, 5K8R, 5DZY, 5I8D, 5DZV, 4ZT1, 2O72, 4ZMY, 4ZI8, 4ZPQ, 5ERP
	EGF-like	5UK5, 5KXH, 5L0V, 5KY0, 1EDM
	Laminin-G like	3SH4, 5IK4, 2JD4, 3PVE, 5MC9
FAT4	Cadherin	5K8R, 4ZPM, 4ZI9, 4ZI8, 4ZPQ, IZXK, 5DZV, 5SZR, 3MVS, 5I8D, 5DZY, 5DZX, 5DZW, 5TPK, 2WCP, 4ZT1, 4ZMY, 4ZPL, 5SZQ, 4OY9, 2O72
	EGF-like	5KY0, 5L0V, 5KY4, 5KXH
	Laminin-G like	2WJS, 5MC9, 3SH4, 5IK4, 2H0B, 5IK8, 2JD4, 1OKQ
ROBO3	Ig-like C2-type	2V9R, 3PUC, 2VRA, 4C4K, 2YD1, 2RJM, 5NOI, 3KNB, 2RIK, 2A38, 2YD1
	Fibronectin type III	1UEM, 4YFE, 4U3H, 4HLJ, 5E7L, 4N5U
CDH23	Cadherin	4AQE, 4AQA, 5DZV,5DZY, 5K8R, 5I8D, 5SZR, 5DZX, 5TPK, 5SZQ, 4ZI8, 4ZPQ, 4ZI9, 4ZPM, 4OY9, 4ZT1, 3PPE, 5CYX, 1ZXK, 2WCP, 3MVS, 4ZMY

Supplemental Table S2. Domain p-values produced in internal control for 296 domains studied in this work. Each row contains a single domain identified by the protein name and domain range, as well as its associated p-value.

Protein	Domain	P-value	Protein	Domain	P-value
CDH23	108-215	1.1181E-03	CFH	19-82	2.06E-02
CDH23	216-328	1.2422E-03	CFH	83-143	7.72E-03
CDH23	329-440	7.2985E-03	CFH	144-207	2.18E-05
CDH23	441-546	3.5300E-06	CFH	208-264	9.57E-04
CDH23	547-650	6.9500E-06	CFH	265-322	8.19E-05
FBN1	81-112	5.0667E-02	CFH	324-386	3.45E-03
FBN1	115-146	1.3007E-02	CFH	387-444	1.12E-03
FBN1	147-178	1.4746E-02	CFH	446-507	5.85E-05
FBN1	184-236	9.5051E-01	CFH	515-566	3.36E-03
FBN1	246-287	2.2768E-03	CFH	567-625	6.25E-07
FBN1	288-329	1.1134E-01	CFH	628-686	8.05E-03
FBN1	334-389	6.3071E-04	CFH	689-746	5.76E-05
FBN1	449-489	2.8309E-03	CFH	751-805	5.23E-06
FBN1	490-529	4.3871E-01	CFH	809-866	1.38E-01
FBN1	530-571	1.4864E-02	CFH	868-928	1.96E-03
FBN1	572-612	5.7299E-01	CFH	929-986	3.40E-03
FBN1	613-653	2.0651E-01	CFH	987-1045	6.45E-04
FBN1	659-711	7.4859E-01	CFH	1046-1104	7.45E-04
FBN1	723-764	6.0900E-05	CFH	1107-1165	1.81E-05
FBN1	765-806	2.9600E-05	CFH	1170-1230	1.02E-02
FBN1	807-846	2.2885E-03	FBN2	111-142	3.05E-02
FBN1	851-902	4.3714E-02	FBN2	145-176	9.83E-03
FBN1	910-951	4.2805E-04	FBN2	176-208	1.30E-02
FBN1	956-1008	2.8755E-03	FBN2	214-266	3.00E-02
FBN1	1028-1069	3.9214E-04	FBN2	276-317	1.88E-02
FBN1	1070-1112	4.7494E-03	FBN2	318-359	7.12E-02
FBN1	1113-1154	5.8231E-02	FBN2	364-417	1.51E-03
FBN1	1155-1196	5.9325E-01	FBN2	494-534	9.43E-02
FBN1	1197-1237	9.6996E-04	FBN2	535-574	2.55E-01
FBN1	1238-1279	3.0613E-04	FBN2	575-616	5.13E-02
FBN1	1280-1321	1.7791E-02	FBN2	617-657	1.01E-04
FBN1	1322-1362	7.0839E-01	FBN2	658-698	1.66E-02
FBN1	1363-1403	1.2938E-03	FBN2	704-756	3.26E-04

FBN1	1404-1445	1.0922E-02	FBN2	768-809	2.44E-03
FBN1	1446-1486	8.0161E-02	FBN2	810-851	4.98E-02
FBN1	1487-1527	3.3544E-03	FBN2	852-891	1.64E-02
FBN1	1532-1589	7.0800E-05	FBN2	896-947	3.29E-03
FBN1	1606-1647	5.3226E-02	FBN2	955-996	2.63E-03
FBN1	1648-1688	4.3107E-02	FBN2	1001-1052	1.26E-03
FBN1	1693-1748	1.4139E-02	FBN2	1073-1114	1.45E-02
FBN1	1766-1807	8.6944E-03	FBN2	1115-1157	1.09E-02
FBN1	1808-1848	9.2035E-04	FBN2	1158-1199	7.37E-01
FBN1	1849-1890	2.6362E-03	FBN2	1200-1241	2.99E-02
FBN1	1891-1929	2.9929E-02	FBN2	1242-1282	3.25E-04
FBN1	1930-1972	1.6640E-02	FBN2	1283-1324	6.22E-05
FBN1	1973-2012	2.1241E-03	FBN2	1325-1366	2.58E-03
FBN1	2013-2054	2.4704E-04	FBN2	1367-1407	1.48E-02
FBN1	2059-2111	1.8290E-02	FBN2	1408-1448	6.00E-02
FBN1	2127-2165	7.1475E-01	FBN2	1449-1490	4.72E-03
FBN1	2166-2205	1.1257E-02	FBN2	1491-1531	1.92E-02
FBN1	2206-2246	4.1755E-03	FBN2	1532-1572	6.66E-03
FBN1	2247-2290	9.8513E-01	FBN2	1577-1633	6.83E-02
FBN1	2291-2332	1.7958E-03	FBN2	1650-1691	2.29E-02
FBN1	2337-2390	3.1966E-02	FBN2	1692-1733	5.24E-03
FBN1	2402-2443	2.5531E-02	FBN2	1738-1791	3.74E-03
FBN1	2444-2484	6.0512E-03	FBN2	1808-1849	3.19E-03
FBN1	2485-2523	1.0709E-02	FBN2	1850-1891	1.03E-02
FBN1	2524-2566	2.2327E-02	FBN2	1892-1933	5.68E-03
FBN1	2567-2606	8.8785E-04	FBN2	1934-1972	2.20E-03
FBN1	2607-2647	5.2547E-02	FBN2	1973-2015	6.39E-02
FBN1	2648-2687	4.7080E-03	FBN2	2016-2055	1.36E-03
EYS	170-212	1.3408E-01	FBN2	2056-2097	2.03E-02
EYS	213-254	2.6126E-04	FBN2	2102-2155	5.92E-04
EYS	256-292	1.4534E-01	FBN2	2171-2212	5.38E-04
EYS	332-368	5.4188E-04	FBN2	2213-2252	2.72E-02
EYS	370-406	1.2068E-03	FBN2	2253-2293	4.97E-05
EYS	567-602	2.2651E-01	FBN2	2294-2337	6.16E-02
EYS	643-679	4.3519E-02	FBN2	2338-2379	2.32E-02
EYS	681-720	2.7479E-02	FBN2	2384-2437	3.61E-02
EYS	733-769	1.6433E-02	FBN2	2449-2490	1.49E-02
EYS	771-807	1.9195E-04	FBN2	2491-2531	4.43E-03

EYS	809-847	6.1003E-04	FBN2	2532-2570	2.56E-02
EYS	849-888	6.6133E-04	FBN2	2571-2613	4.38E-02
EYS	890-926	5.4300E-05	FBN2	2614-2653	3.84E-03
EYS	928-964	1.0919E-01	FBN2	2654-2694	9.68E-02
EYS	966-1002	1.0758E-01	FBN2	2695-2734	9.16E-01
EYS	1004-1040	2.8976E-01	CDH23	34-132	1.09E-04
EYS	1042-1077	5.8944E-02	CDH23	133-236	1.32E-04
EYS	1079-1115	6.7556E-03	CDH23	237-348	2.72E-03
EYS	1117-1159	2.3487E-01	CDH23	349-460	2.88E-03
EYS	1161-1197	4.8390E-02	CDH23	461-561	3.57E-06
EYS	1883-2063	3.2287E-01	CDH23	562-671	4.12E-03
EYS	2099-2140	1.0688E-02	CDH23	672-784	1.11E-02
EYS	2145-2339	5.5125E-02	CDH23	779-890	3.89E-03
EYS	2335-2368	7.4280E-01	CDH23	891-995	4.47E-03
EYS	2371-2408	1.9097E-02	CDH23	996-1102	7.02E-02
EYS	2419-2609	1.3375E-02	CDH23	1103-1208	7.00E-02
EYS	2610-2646	2.0400E-03	CDH23	1210-1313	1.86E-02
EYS	2648-2689	1.3930E-01	CDH23	1314-1418	8.72E-05
EYS	2717-2895	3.4800E-06	CDH23	1420-1527	1.55E-04
EYS	2896-2932	7.4604E-03	CDH23	1529-1634	1.11E-04
EYS	2933-2970	3.5792E-02	CDH23	1635-1744	1.82E-02
EYS	2975-3165	3.3400E-05	CDH23	1745-1851	1.54E-01
FAT4	43-135	3.3956E-02	CDH23	1852-1959	1.69E-03
FAT4	136-250	4.7129E-02	CDH23	1960-2069	2.99E-05
FAT4	251-353	2.6184E-04	CDH23	2070-2174	1.71E-05
FAT4	359-475	2.3010E-03	CDH23	2175-2293	1.87E-02
FAT4	476-582	1.8940E-02	CDH23	2297-2402	8.38E-01
FAT4	584-689	5.3992E-03	CDH23	2403-2509	8.50E-05
FAT4	690-793	2.3112E-04	CDH23	2510-2611	2.23E-03
FAT4	794-893	3.0042E-03	CDH23	2614-2722	6.54E-04
FAT4	894-996	1.1596E-02	CDH23	2729-2846	1.81E-01
FAT4	997-1100	2.4141E-01	CDH23	2847-2975	5.18E-04
FAT4	1101-1210	1.2917E-01	ROBO3	64-160	2.22E-02
FAT4	1211-1315	2.4189E-04	ROBO3	166-253	6.99E-01
FAT4	1316-1420	1.0001E-01	ROBO3	258-342	4.12E-05
FAT4	1421-1529	9.6130E-03	ROBO3	347-440	6.45E-09
FAT4	1529-1629	3.1404E-01	ROBO3	450-531	2.48E-03
FAT4	1630-1740	2.7700E-07	ROBO3	558-652	1.24E-03

FAT4	1741-1841	1.6066E-02	ROBO3	671-766	3.02E-05
FAT4	1842-1944	5.3334E-03	ROBO3	771-869	6.48E-04
FAT4	1945-2051	8.0023E-02	FAT1	35-149	2.47E-03
FAT4	2051-2154	1.3064E-02	FAT1	150-257	3.76E-04
FAT4	2155-2259	3.0624E-04	FAT1	368-463	2.82E-02
FAT4	2260-2364	3.8565E-02	FAT1	464-569	4.08E-07
FAT4	2365-2466	1.3800E-05	FAT1	570-673	1.89E-01
FAT4	2467-2567	1.5100E-03	FAT1	718-822	9.28E-04
FAT4	2568-2669	6.7800E-06	FAT1	823-927	2.16E-02
FAT4	2670-2773	8.0900E-05	FAT1	928-1034	3.63E-03
FAT4	2773-2872	3.6490E-03	FAT1	1035-1139	6.18E-05
FAT4	2873-2983	1.1980E-04	FAT1	1140-1245	5.99E-04
FAT4	2984-3089	1.9959E-02	FAT1	1246-1357	1.31E-02
FAT4	3090-3194	5.4743E-04	FAT1	1359-1456	8.48E-02
FAT4	3195-3298	3.8200E-05	FAT1	1457-1562	3.99E-03
FAT4	3299-3404	2.9400E-05	FAT1	1563-1667	3.92E-04
FAT4	3405-3510	5.1888E-04	FAT1	1668-1765	8.91E-03
FAT4	3509-3620	2.0945E-03	FAT1	1766-1879	4.12E-01
FAT4	3802-3860	1.1606E-01	FAT1	1880-1979	6.92E-02
FAT4	3862-3898	5.6624E-03	FAT1	1980-2081	1.60E-04
FAT4	3900-3936	6.5231E-03	FAT1	2082-2182	2.52E-03
FAT4	3938-3974	7.1505E-02	FAT1	2183-2283	3.37E-02
FAT4	3975-4159	7.8190E-04	FAT1	2284-2390	1.95E-01
FAT4	4162-4198	1.7915E-03	FAT1	2391-2492	2.21E-01
FAT4	4217-4398	1.9200E-05	FAT1	2493-2596	5.42E-02
FAT4	4426-4463	3.5930E-03	FAT1	2597-2703	2.21E-04
Q96QU1	40-147	8.4071E-04	FAT1	2704-2809	6.93E-02
Q96QU1	148-265	1.7600E-05	FAT1	2810-2918	8.87E-04
Q96QU1	278-395	1.6049E-04	FAT1	2919-3023	5.81E-02
Q96QU1	396-509	4.1400E-05	FAT1	3024-3125	4.80E-01
Q96QU1	510-616	1.1032E-01	FAT1	3126-3230	1.96E-04
Q96QU1	617-717	1.0606E-02	FAT1	3231-3335	2.56E-06
Q96QU1	719-819	8.7696E-03	FAT1	3336-3440	4.31E-05
Q96QU1	820-926	8.1110E-03	FAT1	3441-3545	1.50E-02
Q96QU1	927-1035	1.5777E-01	FAT1	3546-3647	1.80E-03
Q96QU1	1037-1144	6.7598E-01	FAT1	3790-3827	9.18E-01
Q96QU1	1145-1259	1.9200E-01	FAT1	3829-4009	9.91E-04
			FAT1	4013-4050	6.43E-02

FAT1	4052-4088	7.03E-03
FAT1	4089-4125	1.26E-03
FAT1	4127-4163	3.80E-01

Supplemental Table S3. Conservation of sequence of 7 domain sets studied. Each domain type (column 1) identifies the number of domains and residues aligned. The third and fourth columns identify the total number of residues which were aligned in the consensus (conserved) and the percentage of these which is identified as critical. The fifth and sixth column identify the total number of residues which were not aligned in the consensus (non-conserved) and the percentage of these which is identified as critical. On average, 52% of all aligned, conserved residues were described as critical while only 34% of non-conserved residues were described as critical.

Domain	Number of domains aligned	Total number of residues aligned	Number of conserved residues	% of critical conserved residues	Number of non-conserved residues	% of critical non-conserved residues
EGF-like	132	5324	2112	41%	3212	25%
TB	9	983	540	45%	443	23%
Cadherin	105	11785	5060	49%	6725	39%
Sushi	20	1187	460	57%	727	43%
Laminin	8	1386	632	56%	754	32%
Fibronectin type-III	3	290	165	62%	125	30%
Ig-like C2-type	5	449	295	56%	154	47%
Average				52%		34%

Supplemental Table S4. Summary of alignment consensus for each domain set. Each domain type can be found in column 1, and for each domain set, the number and types of identically-conserved residues can be found in column 2. The percentage of identically-conserved residues which were discovered to be critical residues is found in column 3. In column 4, the number and types of similarly-conserved residue groups can be found, as well as the corresponding percentage of similarly-conserved residues which were discovered to be critical residues, which can be found in column 5. Columns 6, 7 and 8 contain the average foldabilities of identically-conserved, similarly-conserved, and non-conserved residues. On average, 51% of identically-conserved residues, and 52% of similarly-conserved residues were identified as critical. The average foldabilities of identically-conserved and similarly-conserved residues were 10.04 and 10.36, respectively. The average foldability of non-conserved residues was lower, at 9.10 foldability.

**Domain**	**Frequencies of identically-conserved residues**	**% critical identically-conserved residues**	**Frequencies of similarly-conserved residue groups**	**% critical similarly-conserved residues**	**Average foldability identically-conserved residues**	**Average foldability similarly-conserved residues**	**Average foldability non-conserved residues**
EGF-like	5 Cysteine	40%	7 small (s)<br>2 aromatic (@)<br>1 polar (p)<br>1 tiny (t)	40%	8.8	8.73	8.72
Laminin-G	4 Glycine<br>2 Cysteine<br>1 Serine<br>1 Phenylalanine<br>1 Isoleucine	56%	24 hydrophobic (h)<br>18 polar (p)<br>13 small (s)<br>10 aliphatic (l)<br>4 aromatic (@)	57%	10.41	11.19	9.55
Cadherin	2 Glutamic acid<br>2 Phenylalanine<br>1 Aspartic acid<br>1 Tyrosine	41%	13 aliphatic (l)<br>10 small (s)<br>5 hydrophobic (h)<br>4 polar (p)	48%	9.02	9.84	8.39

	1 Glycine<br>1 Proline		3 charged (c)<br>1 aromatic (@)				
TB	7 Cysteine<br>2 Glycine<br>1 Aspartic acid<br>1 Proline<br>1 Lysine	47%	4 small (s)<br>3 polar (p)<br>3 tiny (t)<br>2 aromatic (@)<br>1 bulky (b)<br>1 aliphatic (l)	43%	9.75	9.44	7.58
Sushi	6 Cysteine<br>1 Tryptophan<br>1 Proline	65%	7 polar (p)<br>4 small (s)<br>3 aromatic (@)<br>3 hydrophobic (h)	55%	11.85	11.01	9.31
Ig-like<br>C2-type	5 Glycine<br>3 Proline<br>2 Cysteine<br>1 Alanine<br>1 Aspartic acid<br>1 Lysine<br>1 Tryptophan	43%	13 hydrophobic (h)<br>11 polar (p)<br>8 small (s)<br>4 aliphatic (l)<br>3 tiny (t)<br>3 charged (c)<br>2 bulky (b)	60%	9.27	11.05	10.37
Fibronectin<br>type-III	2 Glycine<br>1 Tryptophan<br>1 Leucine<br>1 Proline<br>1 Valine<br>1 Alanine<br>1 serine	63%	15 small (s)<br>9 hydrophobic (h)<br>8 polar (p)<br>7 aliphatic (l)<br>3 alcohol (o)<br>2 bulky (b)<br>2 tiny (t)<br>1 aromatic (@)	61%	11.17	11.25	9.77

Average		51%		52%	10.04	10.36	9.1

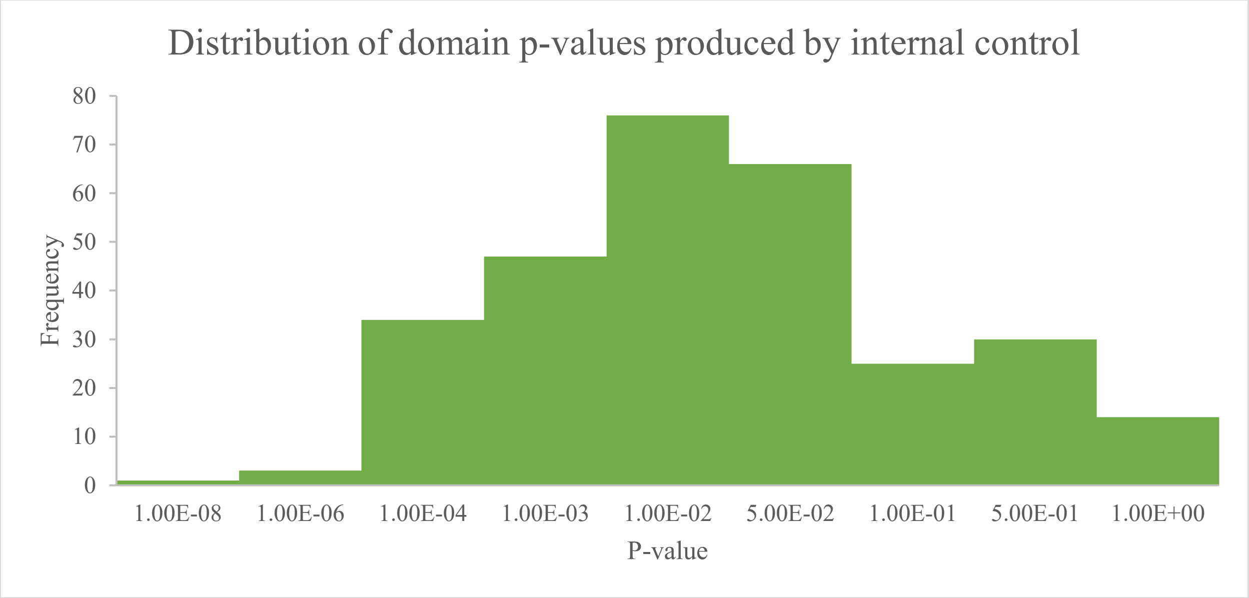

Supplemental Figure S1. Histogram showing the distribution of domain p-values produced by internal control. P-value bins range from magnitudes of 1.00×10^{-8} to 1.00×10^{1}. The distribution shows more than 75% of produced p-values are 0.01 or smaller, and most domain models have p-values centered around 1.00×10^{-2}.

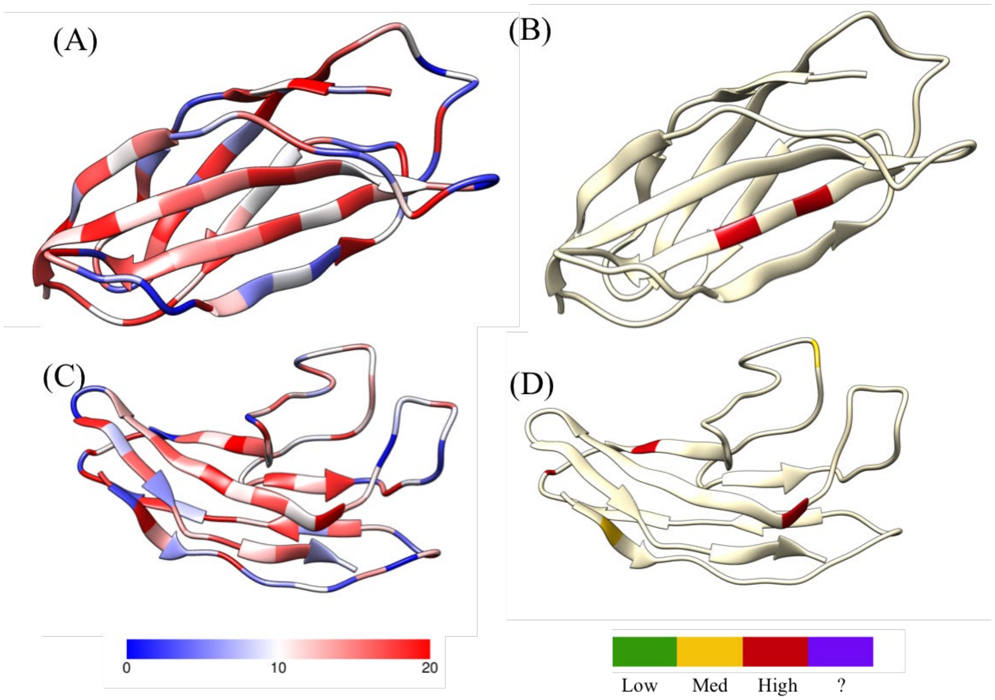

Supplemental Figure S2. Fibronectin type-III and Ig-like C2-type domains of ROBO3 labeled by foldability and HGMD mutations Fibronectin type-III domain (A and B) and Ig-like C2-type domain (C and D) sample of ROBO3 protein colored by foldability (left) and HGMD disease-causing mutations (right). The foldability scale ranges from 0 to 20, with low-foldability residues shown in blue and high-foldability residues shown in red. Tan residues on the right structure are not associated with any disease-causing mutations, while green, yellow, and red residues correspond to low, medium, and high-destabilization mutations. Mutations colored in purple do not have an associated unfolding propensity. 78% of severe mutations of ROBO3 occurred in high-foldability residues.

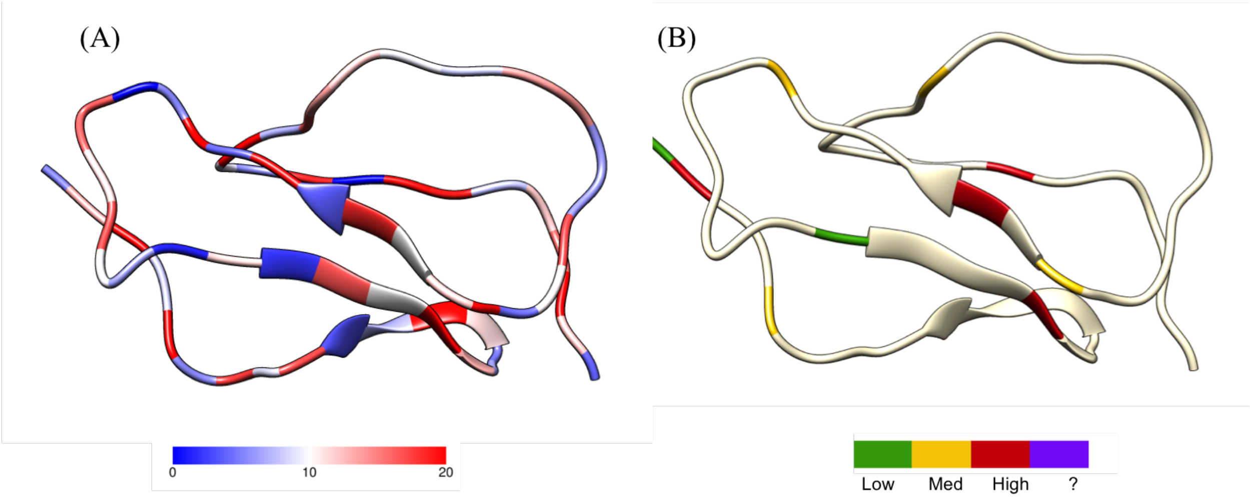

Supplemental Figure S3. Sushi domain of CFH labeled by foldability and HGMD mutations. Sushi domain (A and B) sample of CFH protein colored by foldability (left) and HGMD disease-causing mutations (right). The foldability scale ranges from 0 to 20, with low-foldability residues shown in blue and high-foldability residues shown in red. Tan residues on the right structure are not associated with any disease-causing mutations, while green, yellow, and red residues correspond to low, medium, and high-destabilization mutations. Mutations colored in purple do not have an associated unfolding propensity. 91% of severe mutations of CFH occur in high-foldability residues.

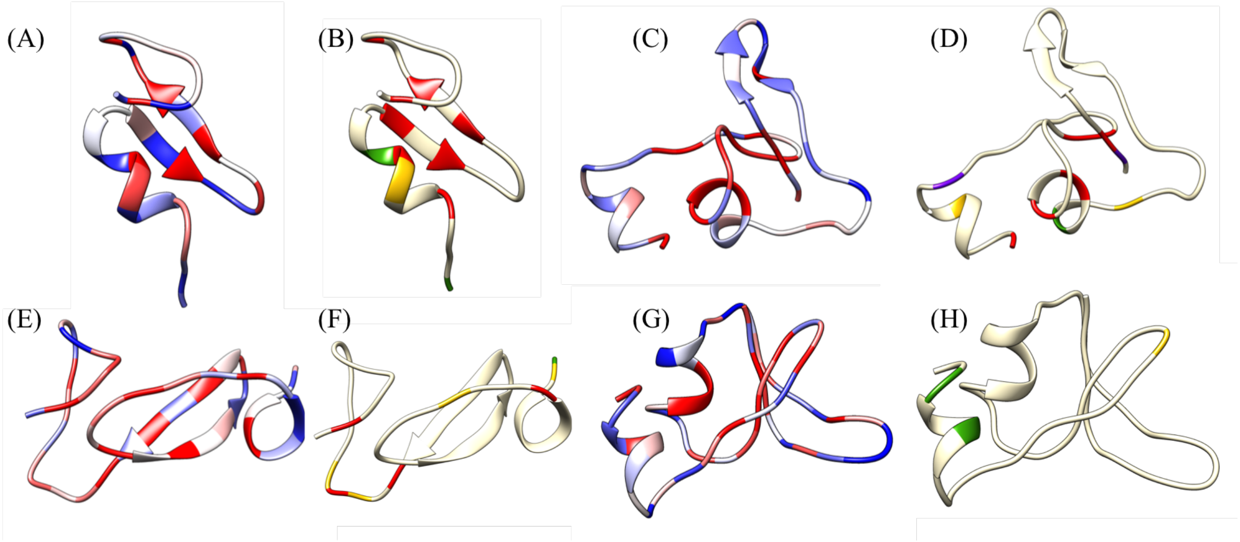

Supplemental Figure S4. EGF-like and TB domains of FBN1 and FBN2 labeled by foldability and HGMD mutations. EGF-like domains (A, B, E, and F) and TB domains (C, D, G, and H) samples of FBN1 (top) and FBN2 (bottom) proteins colored by foldability (A, C, E, and G) and HGMD disease-causing mutations (B, D, F, and H). The foldability scale ranges from 0 to 20, with low-foldability residues shown in blue and high-foldability residues shown in red. Tan residues on the right structure are not associated with any disease-causing mutations, while green, yellow, and red residues correspond to low, medium, and high-destabilization mutations. Mutations colored in purple do not have an associated unfolding propensity. 92% of FBN1 severe mutations occurred in residues which were considered critical. 67% of severe mutations of FBN2 occur in high-foldability residues.

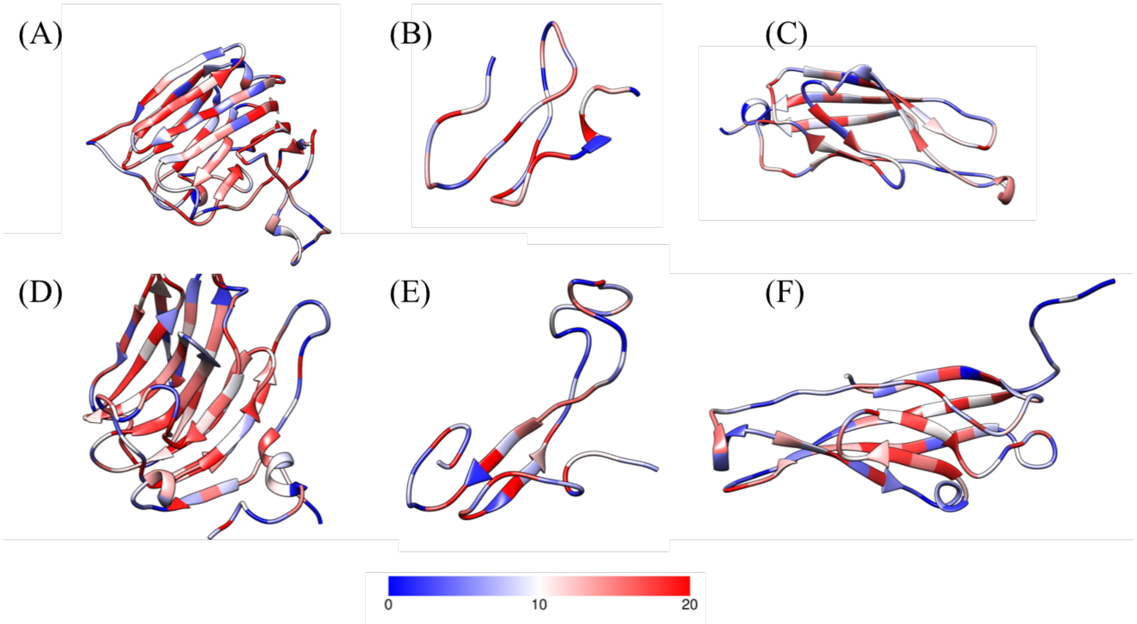

Supplemental Figure S5. Laminin-G, EGF-like, and cadherin domains of FAT1 and FAT4 labeled by foldability. Laminin-G domains (A and D), EGF-like domains (B and E), and cadherin domains (C and F) samples of FAT1(top) and FAT4 (bottom) proteins colored by foldability. The foldability scale ranges from 0 to 20, with low-foldability residues shown in blue and high-foldability residues shown in red. 75% of FAT1 severe mutations occurred in residues which were considered critical. 100% of severe mutations of FAT4 occur in high-foldability residues.

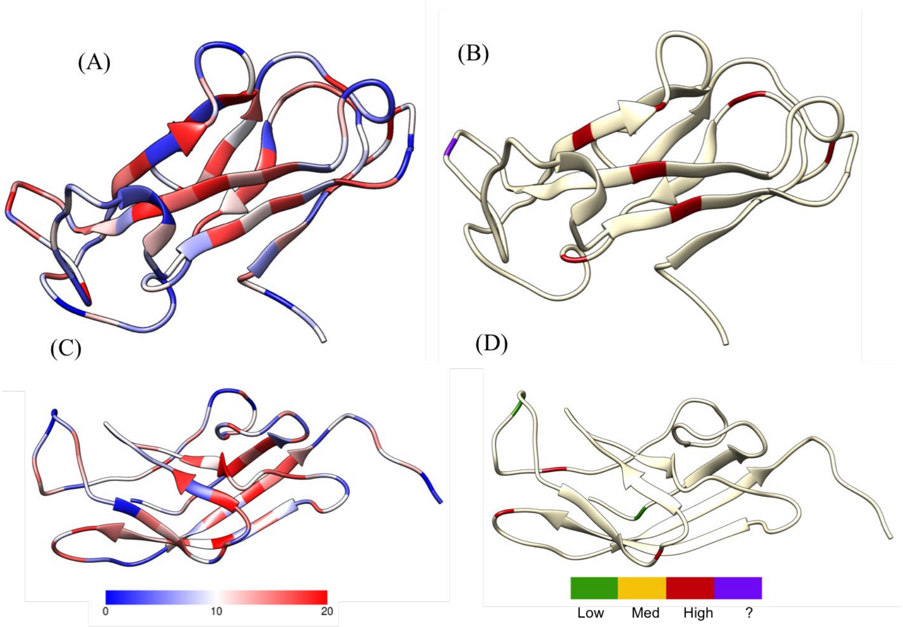

Supplemental Figure S6. Cadherin domains of CDH23 and PCDH15 labeled by foldability and HGMD mutations. Cadherin domains of CDH23(top) and PCDH15 (bottom) proteins colored by foldability (left) and HGMD disease-causing mutations (right). The foldability scale ranges from 0 to 20, with low-foldability residues shown in blue and high-foldability residues shown in red. Tan residues on the right structure are not associated with any disease-causing mutations, while green, yellow, and red residues correspond to low, medium, and high-destabilization mutations. Mutations colored in purple do not have an associated unfolding propensity. 55% of CDH23 severe mutations occurred in residues which were considered critical. 100% of severe mutations of PCDH15 occur in high-foldability residues.

Consensus amino acid symbols:

- **conserved** amino acid residues: **bold and uppercase** letters (such as **G**);
- **aliphatic** residues (**I, V, L**): *l*
- **aromatic** residues (**Y, H, W, F**): *@*
- **hydrophobic** residues (**W, F, Y, M, L, I, V, A, C, T, H**): *h*
- **alcohol** residues (**S, T**): o
- **polar** residues (**D, E, H, K, N, Q, R, S, T**): p
- **tiny** residues (**A, G, C, S**): t
- **small** residues (**A, G, C, S, V, N, D, T, P**): s
- **bulky** residues (**E, F, I, K, L, M, Q, R, W, Y**): b
- **positively charged** residues (**K, R, H**): +
- **negatively charged** residues (**D, E**): -
- **charged** (**D, E, K, R, H**): c

Supplemental Figure S7. Residue groups specified in alignment consensus. The key above identifies symbols for each amino acid group specified in the alignment consensus and specifies which amino acid residues are included in each group.
